# A Review of Lignocellulosic-Derived Nanoparticles for Drug Delivery Applications: Lignin Nanoparticles, Xylan Nanoparticles, and Cellulose Nanocrystals

**DOI:** 10.3390/molecules26030676

**Published:** 2021-01-28

**Authors:** Christian J. Wijaya, Suryadi Ismadji, Setiyo Gunawan

**Affiliations:** 1 Department of Chemical Engineering, Faculty of Industrial Technology and Systems Engineering, Institut Teknologi Sepuluh Nopember, Surabaya 60111, Indonesia; ch.julius7@gmail.com; 2 Department of Chemical Engineering, Widya Mandala Catholic University Surabaya, Kalijudan 37, Surabaya 60114, Indonesia; suryadiismadji@yahoo.com; 3 Department of Chemical Engineering, National Taiwan University of Science and Technology, 43 Keelung Road, Sec 4, Taipei 10607, Taiwan

**Keywords:** cellulose nanocrystals, drug delivery system, lignin nanoparticles, lignocellulosic biomass, xylan nanoparticles

## Abstract

Due to their biocompatibility, biodegradability, and non-toxicity, lignocellulosic-derived nanoparticles are very potential materials for drug carriers in drug delivery applications. There are three main lignocellulosic-derived nanoparticles discussed in this review. First, lignin nanoparticles (LNPs) are an amphiphilic nanoparticle which has versatile interactions toward hydrophilic or hydrophobic drugs. The synthesis methods of LNPs play an important role in this amphiphilic characteristic. Second, xylan nanoparticles (XNPs) are a hemicellulose-derived nanoparticle, where additional pretreatment is needed to obtain a high purity xylan before the synthesis of XNPs. This process is quite long and challenging, but XNPs have a lot of potential as a drug carrier due to their stronger interactions with various drugs. Third, cellulose nanocrystals (CNCs) are a widely exploited nanoparticle, especially in drug delivery applications. CNCs have low cytotoxicity, therefore they are suitable for use as a drug carrier. The research possibilities for these three nanoparticles are still wide and there is potential in drug delivery applications, especially for enhancing their characteristics with further surface modifications adjusted to the drugs.

## 1. Introduction

Drug delivery systems have an important role in medical treatments, especially in carrying drugs to specific targets in the human body. A proper drug delivery system is needed to enhance the effectiveness and efficiency of a disease treatment so that it can significantly affect the healing of a disease. Several methods have been developed and used for drug delivery, such as oral, intravenous, intranasal, transdermal, and nanoparticle-supported methods [1]. Nanoparticle-supported drug delivery systems use various synthetic and natural-derived nanoparticles for storing and delivering various drugs. Excellent results have been generated in the various disease treatments using the nanoparticle-supported method [2]. This method can control the drug release and side effects for a targeted delivery [1,3]. However, the toxicity and deformation of nanoparticles in the human body must be carefully considered. Many synthetic nanoparticles, such as silica nanoparticles [4,5,6] and metal oxides [7,8], can successfully be used as drug carriers, while natural-derived nanoparticles are more potential due to their biocompatibility, biodegradability, and non-toxicity.

Lignocellulosic-derived nanoparticles are a group of nanoparticles derived from natural lignocellulosic biomass containing three main constituents, i.e., 5–30% lignin, 20–35% hemicellulose, and 35–50% cellulose [9]. Lignocellulosic biomass is an abundant resource (approx. 200 billion tons/year) that has much greater potential than is currently being exploited [10]. It can be obtained from various resources, such as wood, grass, agricultural, forestry, industrial, or even household wastes. In the last few decades, there has been a change in the direction of lignocellulosic biomass use from being a fuel source to other advanced applications, such as for chemicals, pharmaceuticals, and advanced materials [9,11]. The use of lignocellulosic biomass, especially from any kind of wastes, is a wise economic consideration. However, the selection of pretreatment is important for an effective additional cost, since a pretreatment is very necessary due to the strong physical and chemical interactions among lignin, hemicellulose, and cellulose [9,12]. A pretreatment is aimed to break the bindings in lignocellulosic biomass for isolating the single pure constituents [13]. Several methods of pretreatment have been established using acids, alkalis, organosolvs, enzymes, ionic liquids, ultrasound-assistant, microwave-assistant, and electrochemical-assistant.

**Figure 1:**
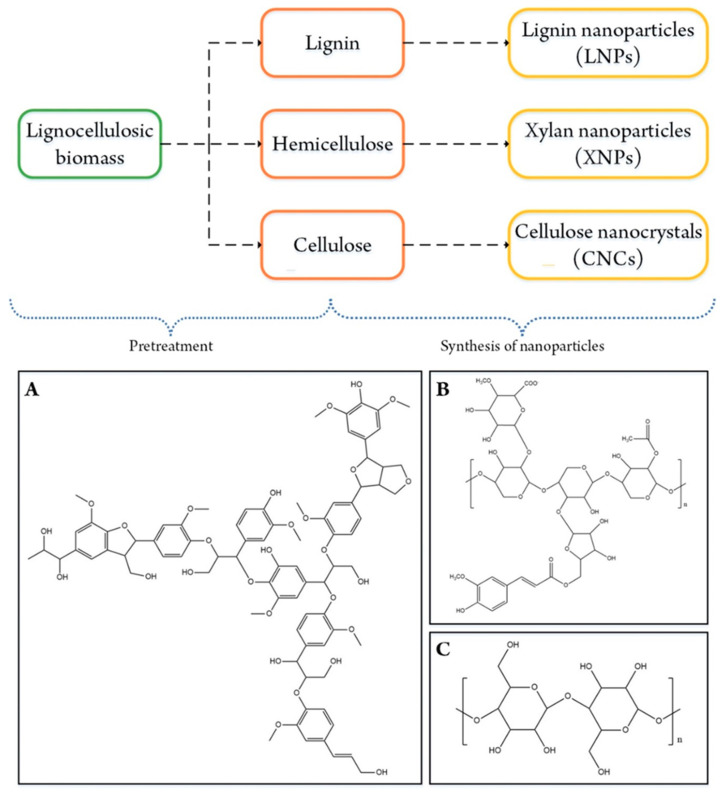
The route of lignocellulosic biomass to lignocellulosic-derived nanoparticles accompanied by the chemical structures of (**A**) lignin, (**B**) hemicellulose, and (**C**) cellulose.

The three major constituents of lignocellulosic biomass, i.e., lignin, hemicellulose, and cellulose, have their respective specialties and advantages. Lignin is an aromatic polymer consisting of syringyl, guaiacyl, and *p*-hydroxyphenyl units [14,15]. The bindings of these three units form groups of *p*-coumaryl, coniferyl, and sinapyl alcohols in a lignin complex structure through covalent bonds [10,15]. Hemicellulose is a branched polymer consisting of xylosyl, arabinosyl, glucoronosyl, glucopyranosyl, and acetyl groups [10,16]. Cellulose is the most abundant biopolymer, which consists of a long homopolymeric chain of glucose units [10,17]. Every lignocellulosic biomass contains different amounts of lignin, hemicellulose, and cellulose which affect the biomass characteristics [11]. For example, agricultural waste has a lower cellulose content than hardwood, but the high lignin content of hardwood provides a strong visible structure. Therefore, the composition and characteristics of lignocellulosic biomass are the main consideration for selecting an appropriate pretreatment method. Due to their superior properties, these three constituents of lignocellulosic biomass can be used as the raw material of lignocellulosic-derived nanoparticles which can further be used in drug delivery applications. There are several types of lignocellulosic-derived nanoparticles that have been widely investigated for drug delivery systems, i.e., lignin nanoparticles (LNPs), xylan nanoparticles (XNPs), and cellulose nanocrystals (CNCs). The route of lignocellulosic biomass for advanced nanoparticles is depicted in Figure 1. In this review, the state-of-the-art overviews of lignocellulosic-derived nanoparticles were provided in terms of drug delivery applications. Herein, the synthesis and characterizations of these lignocellulosic-derived nanoparticles were also discussed, apart from their applications in drug delivery systems.

## 2. Lignin Nanoparticles (LNPs)

### 2.1. Synthesis Methods and Characteristics of LNPs

Lignin nanoparticles (LNPs) are a lignin-derived biopolymer that forms nano-sized structured aggregations. The structure of LNPs is formed by the complicity of the hydrophilic and hydrophobic functional groups of lignin as a precursor. The lignin hydrophobic functional groups are positioned inside the formed aggregation structure and covered by the lignin hydrophilic functional groups, such as phenolic and aliphatic hydroxyl groups [15,18,19]. Hence, LNPs are known as an amphiphilic polymer [19,20], as well as a water-insoluble nanoparticle that is rich in negative surface charge [14,21]. This leads the LNPs to be a good choice as a carrier of a hydrophobic molecule that can be exposed in high-polarity environments [20]. Moreover, LNPs have excellent properties, such as renewability, sustainability, biodegradability, biocompatibility, and safe [14,20,21,22]. Currently, LNPs are mostly applied as binders and carriers in drug delivery, precursors for engineering materials (i.e., carbon fibers and composites), adhesive, emulsifiers, dispersants, templates, and functional materials [14,18]. For drug delivery application, LNPs are suitable due to their biomedical properties, such as antimicrobial, antioxidant, anticancer, anti-inflammatory, UV-blocking, low cytotoxicity, nonhemolytic activity, and less immunogenic [14,20,21,22].

Several synthesis methods have been created and studied for LNPs preparation, such as acid precipitation [23], solvent-shifting [24,25], direct dialysis [21], ultrasonication [14], and homogenization [18]. Table 1 elaborates the advantages and drawbacks of each established method in synthesizing LNPs. The selection of the synthesis method depends on the parameter used and the further application of LNPs. In the acid precipitation method, the types of aqueous acid solution used is the main considerably parameter for adjusting the particle size of LNPs. As shown in Table 2, Agustin et al. [14] investigated the effects of using aqueous hydrochloric acid (HCl), nitric acid (HNO_3_), and sulfuric acid (H_2_SO_4_) solutions toward the particle size of LNPs. The preparations using aqueous HCl and HNO_3_ solutions generated a lower hydrodynamic diameter of LNPs than using aqueous H_2_SO_4_ solution. Moreover, the H_2_SO_4_ precipitated LNPs was unstable in particle size over time because the residual acid on LNPs still initiated the growth of LNPs size [14]. Beisl et al. [23] investigated that the flowrate of lignin solution into the acid solution affected the particle size of LNPs. The smaller size of LNPs could be obtained by increasing the solution flowrate [23]. High supersaturation and mechanical energy increase the collision and aggregation rates so that the formation of larger LNPs size can be avoided.

**Figure 2:**
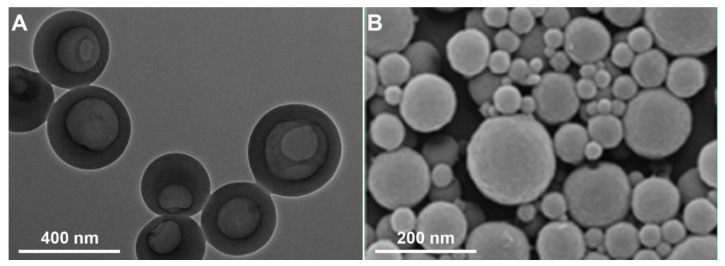
The morphology of LNPs prepared by (**A**) dropping the anti-solvent into the lignin solution (Reprinted with permission from ref [26]. Copyright © 2019 MDPI) and (**B**) dropping the lignin solution into the anti-solvent (Reprinted with permission from ref [18]. Copyright © 2020 American Chemical Society).

In the LNPs’ preparation, solvent-shifting and direct dialysis methods are also classified as self-assembly or nanoprecipitation method, where both are distinguished according to how the solvent and anti-solvent come into contact [15,18,26]. In the solvent-shifting method, the selection of the solvent is important to obtain uniformly sized LNPs. Methanol, ethanol, and tetrahydrofuran have been investigated as the solvent of lignin in the solvent-shifting method [25]. The result showed that the uniformly spherical LNPs were obtained from the lignin solution in methanol, while the ethanol and tetrahydrofuran led to form irregular and heterogeneous particle size of LNPs. The viscosity and interfacial tension of the solvent affected the formation of LNPs during the contact with the anti-solvent [25]. Besides the solvent used, the dropping and stirring rates during the LNPs preparation affects the final particle size of LNPs [27]. The morphology of LNPs is also affected by how the lignin solution in the solvent is contacted with the anti-solvent as shown in Figure 2. Chen et al. [18] obtained the general solid spherical shape of LNPs by dropping the lignin solution into the anti-solvent, while Zhou et al. [26] reported the hollow LNPs by reverse dropping procedure. As compared to the solvent-shifting method, direct dialysis tended to generate around 2.5-fold larger particle size of LNPs [21]. Bertolo et al. [21] reported that LNPs using the direct dialysis method were more hydrophobic than using the solvent-shifting method as shown from the contact angle measurement. In the direct dialysis method, the initial lignin concentration and pre-fractionation step affected the characteristics of LNPs as reported by Lee et al. [28], where the pre-fractionation step differentiated between the lignin factions based on the molecular weight. As shown in Table 2, the increase of the initial lignin concentration generated heterogeneous LNPs with higher particle size and PDI, due to the increased viscosity of initial organic solution [28], while LNPs with smaller to larger particle sizes were produced from the ethanol-, 2-butanone-, and methanol-extracted lignin fractions, respectively [28]. This result indicated that the smaller particle size of LNPs can be obtained from the lower molecular weight-fraction of lignin. However, Lee et al. [28] reported that the organic solvent-extracted lignin fractions generated heterogeneous size distribution (PDI > 0.5). The pre-fractionation step can be used to accomplish the direct dialysis method for producing LNPs, but it must be balanced with careful consideration of the initial lignin concentration.

**Figure 3:**
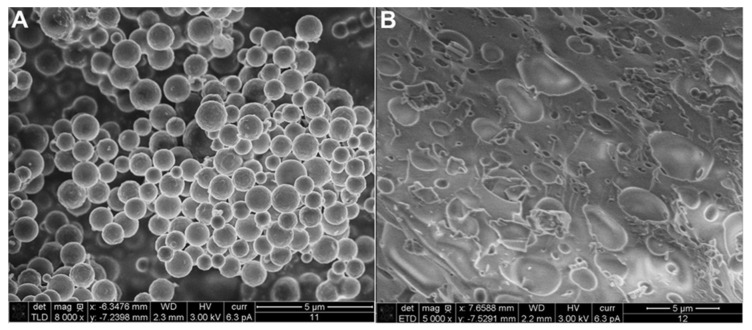
The morphology of LNPs collected by (**A**) freeze-drying and (**B**) centrifugation (Reprinted with permission from ref [29]. Copyright © 2020 Elsevier).

As the LNPs preparation method, ultrasonication and homogenization can be classified as a mechanical treatment method, where the mechanical forces are applied to demolish the lignin into nanoscale size [14]. The ultrasonication method is a time-dependent method as mentioned in Table 1, where the exact processing time is quite difficult to be determined. Herein, longer ultrasonication time may form the radicals due to the over oxidation process, while shorter time exposure of ultrasonic frequencies cannot demolish the lignin into nanoscale size yet [14]. Therefore, short-time ultrasonication is usually only used as a support method to accomplish other methods for generating more homogeneous LNPs. Another mechanical treatment method, homogenization, is solvent-efficient, but high-cost in term of energy consumption (chen2019acs). In the homogenization method, the solvent is the most considerable parameter for collecting appropriate LNPs. Matsakas et al. [19] studied the effect of solvent composition on the particle size of collected LNPs. The higher ethanol composition in aqueous ethanol solution could reduce the particle size of LNPs, but the LNPs became more heterogeneous (higher PDI) and unstable in colloid (lower ζ-potential) [19]. In the next publication of Matsakas et al. [29], the collecting method of LNPs after the synthesis step changed the morphology of LNPs. The amount of exerted force to collect the LNPs could break the shape of LNPs as shown in Figure 3. The high centrifugal force could break the shape of LNPs, so freeze-drying is more recommended to collect the LNPs [29]. Due to the flexibility and ease of control, the solvent-shifting or dropping nanoprecipitation method is more suitable to synthesize LNPs for drug delivery application [18]. This method allows in situ drug encapsulation during the preparation of LNPs, so that high encapsulation efficiency and drug binding. However, the solvent selection needs to be more considered because of the possibility of the solvent left behind due to the absorption in LNPs [18].

**Table 2:** Previous studies of LNPs synthesis using various lignin sources and synthesis methods.

Lignin Sources	Synthesis Methods	Parameters Used	Hydrodynamics Diameters ($D_{H}$) (in nm)	Polydispersity Index (PDI)	ζ-Potential (in mV)	References
Organosolv lignin	Acid precipitation	-	97.3–219.3	-	-	[23]
Alkaline pulping liquor	Acid precipitation + Ultrasonication	HCl as the acid	± 80.0	-	−63 (average value)	[14]
		HNO_3_ as the acid	± 80.0			
		H_2_SO_4_ as the acid	± 90.0			
Enzymatic hydrolysis lignin	Self-assembly	-	286 ± 8	0.208	−38.2 ± 9	[26]
Acid-catalyzed condensed lignin	Solvent-shifting	Aspen wood chips as the raw material	625.0	-	−25 to 15	[15]
		Eucalyptus wood chips as the raw material	468.0			
		Softwood lodgepole pine as the raw material	317.0			
		Corn stover as the raw material	173.0			
Alkali lignin	Solvent-shifting	-	131.2	0.065	−58.2	[25]
Alkali lignin	Solvent-shifting	-	115.9	0.307	−27.0	[21]
	Direct dialysis		300.8	0.298	−25.5	
Organosolv lignin	Solvent-shifting		270.8	0.226	−32.4	
	Direct dialysis		682.3	0.167	−35.1	
Soda lignin	Direct dialysis	1 mg/mL of initial lignin	128.4	0.3	−26.3	[28]
		2 mg/mL of initial lignin	446.7	1.0	−31.8	
		4 mg/mL of initial lignin	559.7	1.0	−37.8	
		Ethyl acetate-extracted lignin	444.8	0.8	−30.6	
		2-butanone-extracted lignin	592.9	1.0	−34.0	
		Methanol-extracted lignin	850.3	1.0	−29.8	
Kraft lignin	Direct dialysis	Waste as the anti-solvent	115.91 ± 6.73	0.265 ± 0.01	−31.99 ± 0.81	[30]
			(0 h storage)	(0 h storage)	(0 h storage)	
			117.15 ± 8.83	0.301 ± 0.03	−32.22 ± 1.09	
			(24 h storage)	(24 h storage)	(24 h storage)	
		Aqueous HCl solution the anti-solvent	152.4 ± 7.21	0.075 ± 0.02	−20.95 ± 1.87	
			(0 h storage)	(0 h storage)	(0 h storage)	
			203.5 ± 5.72	0.212 ± 0.02	−12.01 ± 0.42	
			(24 h storage)	(24 h storage)	(24 h storage)	
		Aqueous HCl solution the initial anti-solvent, and then replaced with water as the final anti-solvent	129.88 ± 4.92	0.175 ± 0.02	−23.71 ± 0.91	
			(0 h storage)	(0 h storage)	(0 h storage)	
			137.6 ± 4.67	0.187 ± 0.02	−23.81 ± 0.75	
			(24 h storage)	(24 h storage)	(24 h storage)	
Alkali lignin	Homogenization	-	∼200.0	0.250	-	[31]
Organosolv lignin	Homogenization	50% *v*/*v* ethanol-water as the solvent	956 ± 10	0.413 ± 0.035	−38.0 ± 1.0	[19]
		75% *v*/*v* ethanol-water as the solvent	530 ± 972	0.502 ± 0.094	−35.4 ± 0.7	
Hybrid organosolv lignin		Water as the solvent	2002 ± 52	0.248 ± 0.016	−47.1 ± 0.6	
		50% *v*/*v* ethanol-water as the solvent	650 ± 9	0.164 ± 0.027	−37.1 ± 1.2	
		75% *v*/*v* ethanol-water as the solvent	488 ± 14	0.486 ± 0.011	−24.5 ± 0.6	

### 2.2. LNPs Application for Drug Delivery System

LNPs have been used as a drug carrier for several drugs, such as curcumin, ovalbumin, resveratrol, benzazulene, sorafenib, doxorubicin, and irinotecan, as summarized in Table 3. The loading capacity and encapsulation efficiency of LNPs become the primary indicator of the LNPs ability in carrying drugs. As shown in Table 3, several studies showed that the loading capacity of LNPs was not too high, but the encapsulation efficiency of LNPs was high enough to indicate that LNPs are suitable, efficient, and effective as a drug carrier. There were two types of drug loading mechanisms onto the LNPs, such as in vitro and ex vitro drug loadings, as studied by Zhou et al. [26]. In vitro drug loading is a mechanism where the drugs are attached to the LNPs during the LNPs preparation step, while the ex vitro mechanism introduces the drugs into the LNPs colloidal solution. The study showed that the doxorubicin loading onto the LNPs with an in vitro mechanism gave a better encapsulation efficiency than the ex vitro mechanism due to the stronger interaction and binding between the drug and LNPs [26]. LNPs have the versatility and strong interactions with various types of drugs, especially with hydrophobic drugs which are the majority of drug characteristics. The interactions between the LNPs and drugs are formed and enhanced by strong hydrogen bonding and π-π stacking due to the similarity of the polyphenolic structure [25,26,27,32]. Aside from that, the negatively charged surface of LNPs carries a special advantage to interact with positively changed drugs through ionic bonding, as has been investigated with ovalbumin-loaded LNPs by Alqahtani et al. [22]. However, Figueiredo et al. [32] reported that capecitabine as a hydrophilic drug was difficult to be loaded onto the LNPs so that the LNPs are more potential for carrying hydrophobic drugs.

The strong interactions between the LNPs and loaded drugs give a slow release characteristic in the drug delivery system [25], which was proven by several previous studies as shown in Table 3. The resveratrol-loaded, irinotecan-loaded, and curcumin-loaded LNPs released over 80% of the loaded drugs at pH 7.4 for 50 h [25], 72 h [30], and 150 h [20], respectively. The drug release from any drug-loaded carriers is a pH-responsive mechanism, which depends on the drug characteristics, carrier characteristics, and interactions between the drug and carrier. As shown in Table 3, many studies proved that the drug-loaded LNPs had a better release and higher amount of released drug at the intestinal condition (pH 7.4) than at pH 2.0 and pH 5.5 [20,30,32]. However, it does not rule out that several drug-loaded LNPs have a better release at lower pH, as mentioned by Zhou et al. [26]. The doxorubicin-loaded LNPs had a higher release at a slightly lower pH (pH 5.5) of the tumor microenvironment [26], which showed the ability of drug-loaded LNPs in targeting more specific release locations. As anti-inflammatory nanoparticles, LNPs can play an important role in the wound healing process. Alqahtani et al. [24] studied empty LNPs treatment, which could fasten the wound closure, where approximately 77% of wounds closed after 12 days. This was faster than untreated wounds and equal to curcumin-treated wounds.

In drug delivery applications, the use of LNPs as a drug carrier should have low cytotoxicity characteristics. Several previous studies have proven this issue, where LNPs had no significant effect on cell viability. Alqahtani et al. [20,24] reported that the cell viabilities of Caco-2 cells and human keratinocyte HaCaT cells after LNPs treatment were above 80% and 98.69% with no significant differences from the untreated cells. Another study investigated the effect of LNPs treatment on several cancer cell lines, such as MDA-MB-231, MCF-7 (breast epithelial cells), PC3-MM2 (prostate epithelial cell), and Caco-2 (colon epithelial cell), and non-tumor cells, such as KG1 (bone marrow macrophage cell) and EA.hy926 (vein endothelial cell) [32]. The cell viability of these cells could be maintained above 80% after 24 h incubation. This proved that LNPs have low cytotoxicity so that only the drugs carried in the LNPs affect the growth of targeted bad cells in the drug delivery system. It can be concluded that the use of LNPs in drug delivery applications is safe and non-toxic. Moreover, the effect of LNPs on KG1 cells indicated no effect of macrophagic activity toward the LNPs, while the study on EA.hy926 cells proved the ability of introduced LNPs in blood vessels [32]. However, the amount of LNPs used and the incubation time need to be considered to be the increase of these parameters could lower the cell viability as investigated by Figueiredo et al. [32].

## 3. Xylan Nanoparticles (XNPs)

### 3.1. Synthesis Methods and Characteristics of XNPs

As a major type of hemicellulose, xylan has great potential for drug delivery application in the form of xylan nanoparticles (XNPs). XNPs are strong nanoparticles that have breaking resistance [33]. XNPs have outstanding properties as other natural-derived nanoparticles, such as biodegradable, biocompatible, non-toxic, non-immunogenic, and antitumor [16,33,34,35]. Due to those properties, XNPs can be used as an adhesive; thickener; emulsifier; stabilizer; additive to plastic, paper, or textile manufacture; magnetite particle carrier; prodrug, and drug carrier; wound dressing; and hydrogels [16,33]. The most common methods for synthesizing the XNPs are precipitation and dialysis methods. In the precipitation method, xylan solution is direct introduced into another certain solution to form the XNPs, such as acid solution [33], ethanol [16], ethyl ether [36,37], or water [38]. The XNPs from the precipitation method tended to have an irregular shape [33,39], while the roughly uniform spherical XNPs could be formed by the dialysis method [34,35,38]. Kumar et al. [39] reported that the average diameter of XNPs reached 103 ± 21 nm, which is suitable as a drug carrier.

In the last few decades, the development of XNPs in various applications is less than other natural-derived nanoparticles. This is due to the complexity of XNPs preparation. An additional step of xylan purification from other hemicellulose-derived monomers is required to produce high-quality XNPs. Moreover, additional steps are necessary to functionalize the XNPs or the drugs in drug delivery application so that the special linkages can be formed. Previous studies have carried out the XNPs or drug functionalizations using several compounds, such as formic acid [33], acetic acid [16], succinic anhydride [34], stearic acid [35,36], 3,3′-dithiodipropionic acid [37], *N*-*N′*-carbonyl diimidazole [35,38], and cholesteryl [40].

### 3.2. XNPs Application for Drug Delivery System

As same as other nanoparticles, XNPs can be combined physically with drugs through an adsorption mechanism or chemically through certain linkages [38]. However, the chemical interaction between XNPs and drugs is usually through ester (covalent) linkages [16], when in general other nanoparticles are through hydrophobic interaction, hydrogen bonding, or π-π stacking. This special characteristic creates a stronger interaction between XNPs and drugs, where it is more acid-resistant than other nanoparticles. XNPs are more suitable to be used as colon-specific drug carrier because these nanoparticles can only be degraded by the colon’s microflora that produces several enzymes, such as β-glucuronidase, β-xylosidase, α-arabinosidase, β-galactosidase, nitroreductase, azoreductase, deaminase, and urea dehydroxylase [16,33]. Due to this unique interaction, an in vitro drug loading mechanism is more appropriate to incorporate the drugs into this biopolymer structure. Previous studies frequently used XNPs as a prodrug carrier, such as 5-aminosalicylic acid (5-ASA) [33], 5-fluorouracil (5-FU) [16], curcumin [34], ibuprofen [38], ketoprofen [35], and doxorubicin [40], where the carried drugs became active drugs after the degradation of XNPs on the targeted site. Kumar et al. [33] and Sauraj et al. [16] investigated the in vitro drug loading of 5-ASA and 5-FU, respectively. The drug loading of 5-ASA into XNPs could reach 511.69 and 665.32 mg/g at the xylan to drug ratio of 1:1 and 2:2, respectively, while it reached 57% and 73% for 5-FU at the same ratio. These proved the quite high loading capacity of XNPs for these two drugs and their dependences on xylan to drug ratio [16,33]. However, another study by Sauraj et al. [36] reported the enhancement of 5-FU drug loading capacity by adding functionalization step of 5-FU using stearic acid.

The degradation of XNPs by colonic enzymes was proved by the studies, and showed that the releases of 5-ASA and 5-FU tended to be non-pH-responsive [16,33]. Only less than 2% of 5-ASA released from the prodrug XNPs at both pH 1.2 and 7.4 [33]. This study was strengthened by the further study by Sauraj et al. [16] that involved the gastrointestinal, cecum, and colon contents of rats for creating more real release environments. The 5-FU could release about 3–4% in gastric condition (pH 4.5), 5–7% in intestine condition (pH 7.4), and 53–61% in the cecum and colonic condition, which were indicating the involvement of the colonic enzymes in degrading XNPs and releasing the drug. However, more recent research are further developing the functionalization of drug-loaded XNPs to enhancing the targeting ability. The curcumin prodrug XNPs had easy breakage of ester linkage due to the high release in acidic conditions [34] so that functionalization is needed to modify and strengthen the structure of prodrug XNPs. Sauraj et al. [36] used stearic acid to enhance the hydrophobic interaction in 5-FU prodrug XNPs, so that the drug released about 28% at pH 7.4 and 58% at pH 5.0 within 60 h without initial burst release. The high enough release at pH 5.0 gives an advantage for the XNPs application in targeting tumor and cancer cells, especially in the colon, where the common pH environment of tumor and cancer cells is about 5.0 [34]. Another functionalization of prodrug XNPs using 3-3′-dithiodipropionic acid has also been carried out to further investigate the targeting ability of functionalized-prodrug XNPs into cancer cells [37]. This functionalization formed the disulfide linkages in the structure of prodrug XNPs to trigger the redox-responsive degradation in cancer cells. This study added glutathione (GSH) into the release medium at pH 7.4 to investigate the effects of the curcumin and 5-FU prodrug XNPs. An outstanding result showed that the high releases (80% of curcumin and 74% of 5-FU) could be obtained by the presence of GSH which caused the breaking of disulfide linkages in XNPs degradation. This promising result improves the likelihood of using XNPs in targeting cancer cells that do contain a high concentration of GSH.

**Figure 4:**
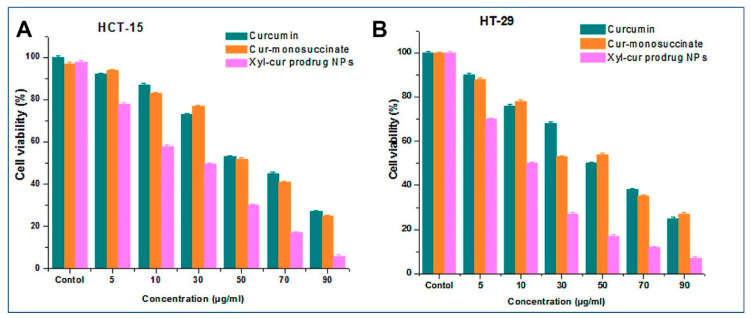
Cytotoxicity of free curcumin, functionalized curcumin, and curcumin prodrug XNPs against colon cancer cells, i.e., (**A**) HCT-15 and (**B**) HT-29 (Reprinted with permission from ref [34]. Copyright © 2018 Elsevier).

In drug delivery application, cytotoxicity is an important assay to test the safety of drugs or their carrier. Previous studies have investigated the cytotoxicity of XNPs and prodrug XNPs. Sauraj et al. [16,34] investigated the cytotoxicity of 5-FU and curcumin prodrug XNPs toward colon cancer cells, such as HCT-116 and HT-29. As shown in Figure 4, the cytotoxicity of curcumin prodrug XNPs was higher than the free curcumin [34], while the same result was showed by the cytotoxicity study using 5-FU prodrug XNPs [16]. These indicated and proved that the xylan itself has antitumor property as said above. Drug-conjugated XNPs enhanced the therapeutic efficacy of cancer cells [16]. Figure 4 shows the reduction of cell viability along with the concentration enhancement. However, their cytotoxicity depended on the dose and the exposure time of prodrug XNPs against cancer cells [34]. 

## 4. Cellulose Nanocrystals (CNCs)

### 4.1. Synthesis Methods and Characteristics of CNCs

Cellulose nanocrystals (CNCs) are a derivative of abundant natural biopolymer, i.e., cellulose, which can be isolated from wood, industrial waste biomass, or agricultural waste. In general, CNCs have rod-like, needle-like, or spherical morphology with the majority of crystalline structure (ca. >85%), where the dimension range of CNCs are 100–3000 nm in length, 3–50 nm in width, and 5–200 in aspect ratio [41]. CNCs can be used for a wide range of applications due to their advanced properties, such as good biodegradability, good biocompatibility, non-toxic, excellent surface charge, and excellent mechanical property [17,42,43]. CNCs have been used in drug delivery systems, beauty and pharmaceutical products, biomedical engineering, shape-memory materials, optical devices, plastics, coatings, gas and energy storages, additives, bio-fillers, catalysts, and food packaging [17,42,43,44]. Due to their pure cellulose content required in the CNCs, raw material selection is very important to be considered for obtaining high-yield CNCs with advanced properties. The raw material must contain high cellulose content which is mostly a crystal region structure. In preparing CNCs from raw natural resources, there are generally four main processes, namely depolymerization, bleaching, isolation, mechanical dispersion, and drying processes, respectively [44]. Depolymerization and bleaching processes are classified and are usually known as pretreatment. Depolymerization is used to break the lignocellulosic chains and degrade the polysaccharide barriers (mostly lignin and hemicellulose) using alkaline, acid, or organosolv solutions, while the bleaching is to completely remove the residual barriers and wash the isolated cellulose. As shown in Table 4, previous studies have used several solutions, such as sodium hypochlorite, sodium chlorite, acetic acid, alkaline, hydrogen peroxide, or combined solutions, to oxidate undesired compounds. The pretreatment process selection depends on cellulose content in raw material, removed components, and raw material characteristics.

In the early development phase of CNCs research, several methods were explored to isolate CNCs from natural resources containing high cellulose content, such as acid hydrolysis, enzymatic hydrolysis, oxidation, steam explosion, subcritical water, ionic liquids, and high-pressure homogenization methods [59,60,61,62,63]. Presently, the acid hydrolysis method is the most widely used method for isolating CNCs due to its simplicity, ease of doing, and desired product characteristics [56]. It needs a shorter processing time compared to the enzymatic hydrolysis method, a lower energy consumption compared to the steam explosion, subcritical water, and high-pressure homogenization methods, and a lower cost compared to the ionic liquid method. Several acid solutions have been investigated as the hydrolysis agent for obtaining high purely crystalline cellulose, such as sulfuric acid [47,54], hydrochloric acid [64], oxalic acid [57], and even combined acid solutions [46]. The penetration of hydronium ion (H_3_O^+^) from the acid solution used into raw cellulose fiber breaks the β-1,4-glycosidic linkages of the amorphous regions of cellulose so that the amorphous cellulose is degraded into its monomers and remained the crystalline region of cellulose [43,48]. The breaks of β-1,4-glycosidic linkages are also occurred in the crystalline region due to the slight diffusion of acid solution into it. However, this only changes the size of crystalline cellulose into nanoscale because the crystalline region is more compact and rigid. Cellulose purity, acid type, acid concentration, processing time, a ratio of solid to the acid solution, and temperature are the important parameters involved in the CNCs preparation using the acid hydrolysis method [44,48]. As mentioned in Table 4, Chieng et al. [51] and Rahman et al. [52] used the same method for CNCs preparation from different cellulose sources, i.e., oil palm mesocarp and tea leaf waste fibers. These research produced CNCs with different sizes and crystallinity indexes due to the cellulose content aspect in the raw materials. Lin et al. [45] investigated the production of CNCs from bleached hardwood pulp using 46 and 63 wt% sulfuric acid hydrolysis, where the products showed a significant difference in size. Xiao et al. [53] reported the effects of processing times (30, 60, and 90 min) toward the characteristics of CNCs, where the increasing hydrolysis time caused the size reduction of CNCs.

The acid hydrolysis method has several drawbacks that needed to be investigated in further research, such as the large amounts of acid solution waste and the residual acid on the CNCs [42,56]. The large amounts of acid solution waste are an environmental issue, especially due to its corrosivity and difficulties to be handled in wastewater treatment. As a further matter besides, the residual acid on CNCs are difficult to be separated and purified. Even though the negatively charged sulphate groups on the CNCs surface increase the colloidal stability, especially when the ζ-potential is lower than −30 mV [43], but it can cause potential disruption in certain applications, such as drug delivery system. In recent research, modified or assisted acid hydrolysis methods were developed to enhance the isolation process of CNCs. Pandi et al. [56] investigated that high-frequency ultrasonication could be used to assist the acid hydrolysis method, where the ultrasound cavitation helped the breakings of amorphous cellulose. This suggested method produced nano-sized CNCs with spherical morphology and high crystallinity index [56]. Herein, high crystallinity plays an important role in terms of mechanical properties, such as rigidity, hardness, and strength [43]. In another study, Lu et al. [57] reported the effectiveness of microwave-ultrasound-assisted acid hydrolysis using molten oxalic acid. The use of sulfuric acid solution could cause excessive hydrolysis process due to its high reactivity so that the product had a lower aspect ratio and yield. However, it could be substituent by molten oxalic acid which has a mild reactivity, so that the processing time was more controllable. Moreover, the assistance of microwave and ultrasound effectively enhanced the degradation of amorphous cellulose.

**Figure 5:**
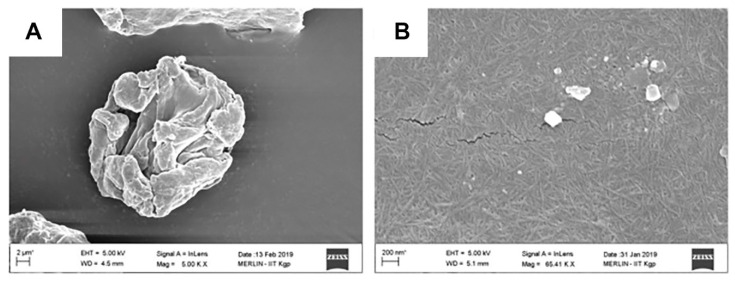
SEM images of CNCs morphologies from (**A**) freeze-drying method and (**B**) drop-casting method (Reprinted with permission from ref [43]. Copyright © 2020 Elsevier).

After isolating CNCs, the supernatant containing CNCs suspension is commonly conducted with mechanical dispersion methods, such as ultrasonication. It is different from before where ultrasonication was used to assist the CNCs production. Here, ultrasonication is used for a produced CNCs suspension to prevent the CNCs agglomeration [58]. Moreover, several studies reported that the size of CNCs changed before and after the ultrasonication process, where it significantly reduced the size of CNCs as shown in Table 4 [43,58]. Furthermore, the drying process plays an important role in collecting solid particles of CNCs. Different drying methods can produce products with different morphologies and characteristics. Prasanna et al. [43] conducted the drying process of CNCs suspension using freeze-drying and drop-casting methods, where Figure 5 showed the morphological differences of dried CNCs. In the freeze-drying method, CNCs tended to agglomerate due to the pre-freezing process, so that the CNCs had an irregular shaped morphology with non-uniform sizes. The controllable CNCs size with a rod-like morphology was able to be obtained by drop-casting method preventing the agglomeration of CNCs particles.

### 4.2. CNCs Application for Drug Delivery System

Due to its biocompatibility, biodegradability, and non-toxicity, CNCs are very potential to be used as a drug carrier in a drug delivery system. Moreover, CNCs are easier to be removed from digestive and bloodstream systems [65], so that its involvements in any human body mechanisms can be prevented and avoided. Higher loading capacity toward drugs may be achieved due to its large surface area and very negative surface charge [66]. Previous studies have been conducted for applying CNCs as the drug carrier of various drugs. CNCs were successfully used as a drug carrier for hydrophilic drugs, such as hydroquinone (HQ) [67], tetracycline hydrochloride (TetHCl) [54,66], procaine hydrochloride (PrHCl), imipramine hydrochloride (ImHCl) [68], and doxorubicin hydrochloride (DoxHCl) [66]. As shown in Table 5, several studied reported that CNCs had good encapsulation efficiencies for ImHCl (85%) and DoxHCl (83%), but mild to low efficiencies for TetHCl (48%), HQ (30%), and PrHCl (20%) [66,67,68]. Hydrophilic drugs can easily bind onto CNCs due to the abundant hydroxyl and carboxyl groups on the surface of CNCs so that CNC tend to have a hydrophilic characteristic [54,67]. Apart from the encapsulation efficiency and loading capacity aspects, the release study is also important to be investigated for knowing the amounts of released drug and the predicted location of drug release in the human body. The human digestive system has various pH conditions for each organ, such as pH 1.2 for gastric fluids and pH 6–7.4 for intestinal or colon fluids [65], so that the pH-dependence release supposes to be studied in investigating a candidate drug carrier material. In Table 5, the cumulative releases of various hydrophilic drug-loaded CNCs were quite high (over 80%), except ImHCl drug whose cumulative release only reached 35% [54,66,67,68]. All of them tended to release between a pH range of 7–7.4 indicating the release in intestinal condition. However, the initial burst release was indicated in the study by Akhlaghi et al. [68], where high amounts of drugs were released rapidly in the early times. This phenomenon showed the drugs used had more tendency into the release medium which affected by pH of the medium, solubility of a drug in the medium, dissociation of the drug, and the strength of drug-CNCs interaction [69].

Recent studies more developed the modification or functionalization of CNCs for enhancing the ability as a drug carrier. Several compounds have been used to modify the surface of CNCs, such as surfactants, small molecule compounds, oils, and polymers [70]. The main aim of surface modification is to change the characteristics of CNCs, especially from hydrophilic to hydrophobic CNCs. It is needed because many drugs have a hydrophobic characteristic, such as therapeutic agents and anticancer drugs. Akhlaghi et al. [68] investigated the use of CNCs modified by TEMPO and chitosan for carrying hydrophilic drugs, but it did not show satisfactory results. This proved that the unmodified CNCs are more suitable to be used as a hydrophilic drug carrier. However, the CNCs modified using rarasaponins gave better loading capacity and release than the unmodified CNCs [71]. A lot of hydroxyl and carboxyl groups were presented on the rarasaponins structure because it was extracted from natural resources (*Sapindus rarak DC*) in the form of crude extract. These functional groups supported the bindings of the hydrophilic drug onto modified CNCs. As described in Table 5, various investigations of CNCs modifications have been done and applied for many hydrophobic drugs, such as paclitaxel [66,72], docetaxel, etoposide [66], luteolin, luteoloside [69], curcumin [70,73,74], and tosufloxacin tosylate [65].

**Table 5:** The capability of CNCs as a drug carrier for several types of drugs.

CNCs or Modified CNCs	Loaded Drugs	Loading Capacity	Encapsulation Efficiency	Release (%)	References
CNCs	Hydroquinone	-	30.0 ± 3%	40 (pH neutral, 1 h)	[67]
				80 (pH neutral, 4 h)	
CNCs	Tetracycline	129.46 mg/g	-	25.1 (pH 2.1)	[54]
				82.21 (pH 7.2)	
CNCs	Procaine HCl	-	±20%	±80 (pH 7.4, 6 min)	[68]
	Imipramine HCl		±85%	±35% (pH 7.4, 2 h)	
TEMPO-CNCs	Procaine HCl		±30%	±60 (pH 7.4, 3 min)	
	Imipramine HCl		±55%	±50 (pH 7.4, 2 h)	
Chitosan-CNCs	Procaine HCl		±20%	±40 (pH 7.4, 12 min)	
	Imipramine HCl		±50%	±80 (pH 7.4, 2 h)	
CNCs	Doxorubicin HCl	-	83%	93 (pH 7.4, 1 day)	[66]
	Tetracycline HCl		48%	87 (pH 7.4, 1 day)	
CTAB-CNCs	Paclitaxel		90%	44 (pH 7.4, 2 days)	
	Docetaxel		90%	59 (pH 7.4, 2 days)	
	Etoposide		48%	75 (pH 7.4, 4 days)	
CTAB-CNCs	Luteolin	12.9 ± 1.5 mg/g	-	44 (pH 6.4, 24 h)	[69]
				57 (pH 7.4, 24 h)	
	Luteoloside	56.9 ± 0.9 mg/g		57 (pH 6.4, 24 h)	
				72 (pH 7.4, 24 h)	
CTAB-CNCs	Curcumin	-	80–90%	-	[73]
CTAB-CNCs	Paclitaxel	65.49 mg/g	87.32%	±25 (pH 5.8, 19 h)	[72]
				±65 (pH 7.4, 19 h)	
SDS-CNCs		43.61 mg/g	59.60%	±65 (pH 5.8, 19 h)	
				±95 (pH 7.4, 19 h)	
Tween 20-CNCs		28.67 mg/g	57.33%	±75 (pH 5.8, 19 h)	
				±80 (pH 7.4, 19 h)	
β-cd-CNCs	Curcumin	8–10%	-	24 (in H_2_O/CHCl_3_, 8 h)	[74]
l-l-MA-CNCs	Tosufloxacin tosylate	29.14%	99.84%	40.38 (pH 7.4, 30 h, without enzyme lysozyme)	[65]
				72.55 (pH 7.4, 30 h, with enzyme lysozyme)	
RS-CNCs	Curcumin	12.40 ± 0.24% (at 10 h)	49.49 ± 0.94% (at 10 h)	43 (pH 7.4, 1 day)	[70]
				78 (pH 7.4, 3 days)	
RS-CNCs	Tetracycline	13.97 mg/g (only CNCs) 18.11 mg/g (modified CNCs)	-	18.28 (pH 3, 14 h)	[71]
				55.49 (pH 7, 14 h)	

Note: TEMPO–2,2,6,6-tetramethylpiperidine-1-oxyl radical; CTAB–cetyltrimethylammonium bromide; SDS–sodium dodecyl sulfate; β-cd–β-cyclodextrins; l-l–l-leucine; MA–maleic anhydride.

Putro et al. [72] studied the effects of CNCs modifications using cationic, anionic, and nonionic surfactants in hydrophobic drug delivery. Due to the negative surface charge of CNCs, a cationic surfactant had good bindings onto CNCs [69,72]. It was proved by the higher loading capacity (65.49 mg/g) and encapsulation efficiency (87.32%) of the cationic CNCs than the anionic and nonionic CNCs [72]. The modification of CNCs using various compounds is very potential for enhancing the ability of CNCs in drug delivery application. Several techniques have been established for modifying the surface of materials, such as oxidation, sulfonation, carboxylation, esterification, silylation, cationization, and grafting [71]. However, the selection of modifying agents is needed to be considered carefully in case of health and environmental issues. As an example, rarasaponins was chosen as a modifying agent for CNCs due to its safety for health and environment, where it is biodegradable and non-toxic surfactant from natural resources [70,71].

Due to the increased research interest in CNCs production, especially in drug delivery applications, the potential for toxicity is needed to be known and investigated at least using antibacterial activity or cytotoxicity. It is important to the effects of nanoparticle exposure in living cells for confirming nanoparticle safety [47]. In terms of toxicity, the size, shape, surface area, and degradation or destruction products of nanoparticles are potential aspects in the toxicity behavior [47,49]. Several studies of CNCs cytotoxicity were carried out using Caco-2 cells (human colon epithelial cells) [47,53], A549 cells (human lung epithelial cells) [49], and 7F2 cells (normal mouse bone marrow osteoblast cells) [72]. In the assessment using Caco-2 cells, Coelho et al. [47] and Xiao et al. [53] reported the same high viabilities of Caco-2 cells treated with CNCs in the culture medium, where it indicated the low cytotoxicity of CNCs and its safety in drug delivery application. Based on the cytotoxicity assay, the maximum concentration of CNCs can be treated to Caco-2 cells was 2 mg/mL with cell viability of 93.11%, where the viability of Caco-2 cells treated with higher concentrations decreased significantly [53]. On another cytotoxicity assay, spherical CNCs produced from *Ferula gummosa* roots gave lower viabilities (70–30%) of A549 cells for a wide variety of CNCs concentrations [49]. Even though, the spherical and rod-like nanoparticles have lower potential damage on living cells than the needle-like shape. However, the size of CNCs was a potential cause of CNCs cytotoxicity, where the produced CNCs had a much smaller size of 22.11 ± 5 nm compared to the other CNCs from any variety of raw materials [49]. As compared with the cytotoxicity assay on diseased cells, Putro et al. [72] has investigated the cytotoxicity of unmodified and modified CNCs on normal cells (7F2 cells). Here, unmodified CNCs had high cell viability, but modified gave various levels of cell viability. In detail, SDS-CNCs and Tween 20-CNCs had slightly lower viabilities, but CTAB-CNCs showed bad cell viabilities (< 40%) [72]. These viabilities got even lower along with the increase of CNCs concentration in the culture medium. Therefore, the use and selection of modifying agents must be considered carefully due to their high impact on the characteristic of CNCs.

## 5. Comparisons of LNPs, XNPs, and CNCs in Drug Delivery Application

In general, these three nanoparticles have similar excellences for drug delivery application, such as biodegradable, biocompatible, safe, and low cytotoxicity. These advantages are possessed because these nanoparticles are synthesized from natural resources. More than that, these nanoparticles have controlled and pH-responsive release capability which is one of the important aspects of a drug delivery system. However, each LNPs, XNPs, and CNCs have specific advantages and disadvantages in drug delivery application, as described in Table 6. The uses of LNPs, XNPs, and CNCs have to be adjusted with several considerations, such as loaded drug characteristics, nanoparticle-drug interactions, release mechanism, and targeted release location. Here, the excellences of nanoparticles are affected by the precursor’s characteristics and synthesis methods. In Table 6, an amphiphilic characteristic of LNPs comes from the hydrophilic and hydrophobic tails of lignin, while the ability of LNPs and XNPs in wound treatment is due to the anti-inflammation characteristic of their precursors. In another case, the acid involvement in the synthesis process gives negative surface charges on the LNPs and CNCs. Besides that, these nanoparticles still have several weaknesses that can affect the drug delivery mechanism. The functionalization of nanoparticles using a certain modifying agent can overcome the weaknesses and improve the characteristics. Recent studies on LNPs, XNPs, and CNCs tend toward nanoparticle functionalization for overcoming the weaknesses.

## 6. Conclusions and Future Perspectives

Lignocellulosic-derived nanoparticles, i.e., LNPs, XNPs, and CNCs, are successfully synthesized using various methods, but one or more challenging pretreatments are needed to obtain a high purity constituent. The raw material characteristics, pretreated-constituent purity, synthesis methods, synthesis parameters are necessarily considered and adjusted to obtain the desired lignocellulosic-derived nanoparticles. Although the synthesis process is quite challenging, it is commensurate with the privileges of the nanoparticles, such as biocompatibility, biodegradability, and non-toxicity. Moreover, the strong physical and chemical interactions between nanoparticles and drugs are provided for their drug delivery applications, so that the drug release can be controlled and targeted to specific locations in the human body. In the future, the research of lignocellulosic-derived nanoparticles for drug delivery applications is wide open. The surface modification of nanoparticles is needed for enhancing the ability of nanoparticles attached by various drugs. Furthermore, more clinical trials are necessarily investigated for drug-loaded nanoparticles to provide biomedical and behavioral studies in the human body.

## Figures and Tables

**Table 1:** Advantages and drawbacks of various LNPs synthesis methods.

Methods	Descriptions	Advantages	Drawbacks	References
Acid precipitation	A lignin solution (in alkali or ethylene glycol) is gradually added by an acid solution	Monodisperse size distribution	Existence of residual acid pH-responsive	[14,23]
Solvent-shifting or dropping nano-precipitation	A lignin solution in the organic solvent (such as tetrahydrofuran, dimethyl sulfoxide, or dioxane) is introduced into an anti-solvent (such as water)	Simple process Cost-effective process Scale-up possibility	Long-time process Heterogeneous size distribution High solvent consumption Solvent-type-dependent process Existence of residual solvent	[14,18,21,24,25]
Direct dialysis or dialysis nano-precipitation	A lignin solution in the organic solvent is dialyzed using a dialysis cellulose membrane against water	Simple process Cost-effective process Less independent parameters	Larger-sized nanoparticles Long-time process	[21]
Ultrasonication	A lignin solution in water is sonicated	Simple process No toxic organic solvent uses	Time-dependent process	[14]
Homogenization	A lignin solution in water is processed in a homogenizer	No organic solvent uses (only water) Simple and straightforward process	High energy consumption	[18,19]

**Table 3:** The capability of LNPs as a drug carrier for several types of drugs.

Loaded Drugs	Loading Capacity (%)	Encapsulation Efficiency (%)	Release (%)	References
Curcumin	-	92 ± 4	34 (intestinal pH 7.4; 8 h)	[20]
			±80 (intestinal pH 7.4; 150 h)	
			8.7 (gastric pH 2; 4 h)	
			±25 (gastric pH 2; 150 h)	
Ovalbumin	-	81.64		16.4 ± 4.2 (pH 7.4; 12 h)
Resveratrol	>20	> 90		> 80 (pH 7.4; 50 h)
Benzazulene	8 ± 1	77 ± 10	90 (pH 5.5; 6 h)	[32]
			95 (pH 7.4; 6 h)	
Sorafenib	7 ± 2	68 ± 19	61 (pH 5.5; 6 h)	
			100 (pH 7.4; 6 h)	
Doxorubicin	-	67.5 ± 6 (In vitro)	21.3 (pH 5.5; 4 h)	[26]
		49.4 ± 7 (Ex vitro)	15.2 (pH 7.4; 4 h)	
Doxorubicin	>12.5	>60	>50 (pH 5.5; 60 h)	[27]
			>65 (pH 7.4; 60 h)	
Irinotecan	13.61 ± 0.59	67.65 ± 1.95	22.11 ± 4.05 (pH 7.4; 2 h)	[30]
			43.84 ± 6.07 (pH 7.4; 8 h)	
			86.72 ± 7.05 (pH 7.4 72 h)	

**Table 4:** Previous studies of CNCs preparation using various raw materials and methods.

Raw Materials	Preparation Methods		Crystallinity Index (in %)	Morphology	Particle Size (in nm)	ζ-Potential (in mV)	References
	Pretreatment	Treatment					
Bleached hardwood pulp	Alkaline pretreatment	Acid hydrolysis (46 & 63 wt% H_2_SO_4_)	70–80	Rod- or needle-like shape	±600 (46 wt% H_2_SO_4_)	-	[45]
					±250 (63 wt% H_2_SO_4_)		
Cucumber peels	Hot water, acid, and alkaline pretreatments + NaOCl bleaching	Acid hydrolysis (60 wt% H_2_SO_4_)	74.1	Irregularly shaped flakes (freeze-drying method) and rod-like shape (drop-casting method)	582.96 (without sonication)	−48.4 ± 1.3	[43]
					110.9 (with sonication)		
Seaweed	Acid and alkaline pretreatments + NaOCl & H_2_O_2_ bleaching	Acid hydrolysis (51 wt% H_2_SO_4_)	98.89 ± 0.24	Rod-like shape	239.43 ± 38.57 (length)	-	[44]
					22.45 ± 6.51 (width)		
Cashew tree pruning residue	Acetosolv pretreatment + alkaline-peroxide bleaching	Acid hydrolysis (60 vol% + 25 vol% HCl)	-	Needle-like shape	276 ± 45.7 (length)	−26.1 ± 2.4	[46]
					17.5 ± 4.52 (width)		
Grape pomace	Organosolv, acid, alkaline pretreatments + alkaline-peroxide bleaching	Acid hydrolysis (64–65 wt% H_2_SO_4_)	74.89	Needle-like shape	323 (length)	-	[47,48]
					7 (width)		
*Ferula gummosa* roots	Alkaline pretreatment	Acid hydrolysis (64 wt% H_2_SO_4_)	84.01	Spherical shape		22.11 ± 5	[49]
Cornstalk	Organosolv extraction + acid and alkaline pretreatments	Acid hydrolysis (60 wt% H_2_SO_4_)	69.20	Needle-like shape	120.2 ± 61.3 (length)	-	[50]
					6.4 ± 3.1 (width)		
Oil palm mesocarp fibers	Alkaline pretreatment + acetate buffer-NaClO_2_-water bleaching	Acid hydrolysis (65 w% H_2_SO_4_)	77.80	Rod-like shape	4.52 (width)	-	[51]
Tea leaf waste fibers	Alkaline pretreatment + acetate buffer-NaClO_2_-water bleaching	Acid hydrolysis (65 w% H_2_SO_4_)	83.1	Rod-like shape	7.97 (width)	-	[52]
Wheat bran	Organosolv, enzymatic, and alkaline pretreatments + NaClO_2_ bleaching	Acid hydrolysis (64 wt% H_2_SO_4_)	66.67 (30 min)	Needle-like shape	644.77 ± 225.20 (30 min)	−36.5 ± 0.8 (30 min)	[53]
			70.32 (60 min)		568.81 ± 229.66 (60 min)	−39.8 ± 1.0 (60 min)	
			66.74 (30 min)		486.18 ± 177.36 (30 min)	−39.6 ± 1.2 (30 min)	
Passion fruit peels waste	Alkaline pretreatment + alkaline-peroxide bleaching	Acid hydrolysis (52 wt% H_2_SO_4_)	77.96	Rod-like shape	103–173.5	−25 to −22	[54]
Bamboo shoots	Alkaline pretreatment + alkaline-peroxide bleaching	Acid hydrolysis (55 wt% H_2_SO_4_)	83.65	Rod-like shape	-	-	[55]
Waste cotton from hospital	NaOCl bleaching	Ultrasound-assisted acid hydrolysis (50 wt% H_2_SO_4_)	81.23	Spherical shape	221	-	[56]
Dissolving bamboo pulp	-	Microwave-ultrasound-assisted acid hydrolysis (oxalic acid)	78.31	Rod-like shape	285 (length) 17 (width)	−42.9	[57]
Bleached *Eucalyptus* Kraft pulp	-	Acid hydrolysis (62 wt% H_2_SO_4_)	90.3 ± 0.0	Needle-like shape	<10 (width)	-	[58]
		Enzymatic hydrolysis using Cellic CTec 2 (Novozymes)	94.1 ± 2.7	Needle-like shape	6–12 (width)		
Sugarcane bagasse	Steam explosion + alkaline pretreatment + alkaline-peroxide bleaching	Enzymatic hydrolysis using Cellic CTec 2 (Novozymes)	96.5 ± 1.1	Needle-like shape	14–22 (width)		
Cotton cellulose powder	Alkaline pretreatment + NaClO_2_ bleaching	High-pressure homogenization	79 ± 1	Needle-like shape	177.5 ± 123.7 (length)	-	[59]
					7.7 ± 3.0 (width)		
Microcrystalline cellulose	-	Subcritical water	79.0	Rod-like shape	242 ± 98 (length)	-	[60]
					55 ± 20 (width)		
Eucalyptus bleached hardwood Kraft pulp	-	Ionic liquids treatment	-	Spherical shape	123 ± 48 ([bmim][HSO_4_])	−24 ± 2.5 ([bmim][HSO_4_])	[61]
					77 ± 25 ([emim][Cl])	−12 ± 6 ([emim][Cl])	
Crown flower	Alkaline pretreatment + acetic acid-H_2_O_2_ bleaching	Solid acid-catalyzed exfoliation	43.02	Rod-like shape	242.06 ± 80.79 (length)	−15.6 ± 1.4	[42]
					8.80 ± 2.92 (width)		

**Table 6:** The excellences and weaknesses of LNPs, XNPs, and CNCs for drug delivery application.

Nanoparticles	Excellences	Weaknesses
LNPs	Amphiphilic nanoparticles (tend to be hydrophobic) Negatively charged nanoparticles Strong hydrogen bonding and π-π stacking interaction with various drugs Ability in wound treatment	Lower drug loading capacity Less ability in hydrophilic drug carrier
XNPs	Hydrophilic nanoparticles Breaking- and acid-resistant nanoparticles Ester (covalent) linkage with various drugs Ability in wound treatment	Less strong interaction with various drugs Degradation only by colon’s microflora Less ability in hydrophobic drug delivery
CNCs	Hydrophilic nanoparticles Negatively charged nanoparticles Strong hydrogen bonding and π-π stacking interaction with various drugs High drug loading capacity	Less ability in hydrophobic drug delivery

## Data Availability

The data presented in this study are openly available and cited in the references.
